# Flow Behavior of AA5005 Alloy at High Temperature and Low Strain Rate Based on Arrhenius-Type Equation and Back Propagation Artificial Neural Network (BP-ANN) Model

**DOI:** 10.3390/ma15113788

**Published:** 2022-05-26

**Authors:** Sijia Li, Wenning Chen, Krishna Singh Bhandari, Dong Won Jung, Xuewen Chen

**Affiliations:** 1 Department of Mechanical Engineering, Jeju National University, Jeju-si 63243, Korea; lisjxy98@stu.jejunu.ac.kr (S.L.); chenwnxy98@stu.jejunu.ac.kr (W.C.); Krishna.bhandari51@gmail.com (K.S.B.); 2 School of Materials Science and Engineering, Henan University of Science and Technology, 263 Kaiyuan Avenue, Luoyang 471023, China

**Keywords:** AA5005 alloy, high temperature, Arrhenius-type, flow stress, low strain rate, BP-ANN, constitutive relationship

## Abstract

To realize the purpose of energy saving, materials with high weight are replaced by low-weight materials with eligible mechanical properties in all kinds of fields. Therefore, conducting research works on lightweight materials under specified work conditions is extremely important and profound. To understand the relationship of aluminum alloy AA5005 among flow stress, true strain, strain rate, and deformation temperature, hot isothermal tensile tests were conducted within the strain rate range 0.0003–0.03 s^−1^ and temperature range 633–773 K. Based on the true stress-true strain curves obtained from the experiment, a traditional constitutive regression Arrhenius-type equation was utilized to regress flow behaviors. Meanwhile, the Arrhenius-type equation was optimized by a sixth-order polynomial function for compensating strain. Thereafter, a back propagation artificial neural network (BP-ANN) model based on supervised machine learning was also employed to regress and predict flow stress in diverse deform conditions. Ultimately, by introducing statistical analyses correlation coefficient (R^2^), average absolute relative error (AARE), and relative error (δ) to the comparative study, it was found that the Arrhenius-type equation will lose accuracy in cases of high stress. Additionally, owning higher R^2^, lower AARE, and more concentrative δ value distribution, the BP-ANN model is superior in regressing and predicting than the Arrhenius-type constitutive equation.

## 1. Introduction

Possessing excessive weight and mechanical properties, most traditional industrial products consume immense amounts of unnecessary energy. To cater to the worldwide tendency of energy-saving, promise strategies were put forward (e.g., renewable energy, machine updating, and light material application). Substituting traditional high-weight materials like carbon steel with lightweight materials such as aluminum alloy, titanium alloy, and magnesium alloy, light material application is regarded as one idea with excellent feasibility. At the same time, these light materials need to guarantee satisfactory mechanical performance. Investigation from the auto industry reveals that 8% of energy consumption can be saved by paring down 10% of vehicle weight [1]. Hence, the mechanical properties of light materials must be investigated concretely and credibly before application. As a light material with good corrosion resistance [2], AA5005 alloy is widely used in conductors, cookers, auto interior materials [3], etc., and has a great application potential. However, because of the characteristics of medium strength and temperature and strain rate sensitivity, the applications of Al AA5005 are limited by its poor formability at room temperature and medium-high speed deformation conditions [4]. Consequently, aiming at providing a detailed flow behavior reference to the work of improving the formability of AA5005 alloy, research on studying flow stress behaviors at high temperatures and low strain rates of this material is vital and meaningful.

In conventional ways, regression constitutive equations were extensively used to regress the flow stress curves to describe materials’ flow behavior. Two main kinds of equations were divided in the process of developing constitutive relations: theoretical constitutive equations based on micro deformation mechanisms and phenomenal constitutive equations based on macro experimental data. However, due to the characteristics that are complex to solve as too many parameters are needed, it is difficult to apply theoretical constitutive equations to engineering. Conversely, owing to the merits of fewer parameters, simple forms, and adequate accuracy, phenomenal constitutive equations are extensively adopted in research and engineering. So far, many phenomenal constitutive equations that comprehensively consider the effects of strain rate, deformation temperature, and strain during deformation processes have been presented. Among these equations, the Johnson-Cook equation [5], Fields-Backofen equation [6], Zerilli-Armstrong equation [7], and Arrhenius-type equation [8,9] are the most famed. The Arrhenius-type equation was adopted in this work due to its high accuracy in modeling aluminum alloys [10].

Numerous works were undertaken by researchers with Arrhenius-type constitutive equations in multifarious materials and various working conditions. In most cases, the equation accuracy will increase with the increase in the number of test sets [11,12]. Still, a larger deviation can be seen under the condition of high stress [12,13]. Additionally, optimization works related to the Arrhenius equation were also conducted in in-depth research. A new idea that combines regression analysis with an iterative method was raised by Wang et al. [14] to express a magnesium alloy flow stress behavior; after a series of isothermal compression tests, good results were achieved. Similarly, by introducing the concept of reduced gradient refinement to the Arrhenius equation, Bodunrin [15] successfully reduced 30–40% AAREs of two titanium alloys. So far, the AAREs calculated by the Arrhenius-type constitutive equation have always been in the range of 3–8%, as well as its modification methods [11,12,13,14,15].

Nevertheless, material deformation is a complex process with extremely high non-linear problems, changes, or fluctuations. During the deformation process, arbitrary parameter change will result in increasing error, especially at a high strain rate [11,13]. In addition, the constitutive equation only takes effect on the materials without phase region transformation in most conditions. New parameters need to be determined, and the equation needs to be recalculated when the phase region changes. All these factors are limitations of constitutive regression equations. Since the purposes of flow behavior models are to deliver references to formulate reasonable forming schemes and to provide models to conduct numerical simulation, high model accuracy is the unremitting pursuit of all researchers. Thus, new methods need to be explored to break the limitations of constitutive equations. Fortunately, machine learning has been increasingly adopted to solve classification and regression in all kinds of fields. Based on imitating the way human neurons process information, artificial neural networks (ANNs) can deal with complicated non-linear problems. Above all, high non-linear problems caused by internal metallurgical transformation can be avoided with this technology. One of the most used ANNs is the BP-ANN, which was firstly proposed by Werbos P [16].

So far, many flow stress regression studies have been undertaken with ANNs and the results’ accuracy has been well verified. Bobbili et al. [17] obtained high accuracy results for the Johnson-Cook equation under high-speed working conditions of the armor steel. For the compressive deformations involving creak and instability, constitutive equations always deliver poor predictability. A comparative study was carried out, and indicated that the ANN model can achieve high-precision outcomes without considering instability influence [18]. Furthermore, the ANN model has better describing capability than the Arrhenius-type equation, which was verified under the same procedure when oscillations happen on the curves [19]. Isothermal hot compression tests were carried out by Ji et al. [20] and the results declare that the predicting ability of the BP-ANN model is not affected by interconnecting metallurgic phenomena while maintaining high accuracy, which is opposite to the Arrhenius-type model.

Although numerous works have been conducted with regression equations to describe flow stress with all kinds of materials, AA5005 alloy has not been reported. Moreover, there are few researches that have studied the aluminum low strain rate deformation. In the present study, isothermal tensile tests were accomplished within a low strain rate range of 0.0003–0.03 s^−1^ and a deformation temperature range of 633–773 K. Then, the strain-compensated Arrhenius-type equation and the BP-ANN model were adopted to regress the flow stress behavior from the perspective of performance. The detailed calculation processes of the two models were introduced. Finally, assisted by statistical analysis methods like R^2^, AARE and δ, a comparative study was conducted to determine the predictability of both models. The developed models lay an application foundation for process design and simulation of AA5005 alloy.

## 2. Materials and Methods

### 2.1. Experimental Procedures

**Figure 1:**
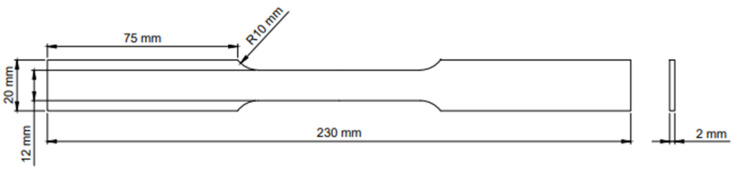
Dimensions of the specimen.

The AA5005 alloy chemical composition (wt.%) is Mg 0.97, Fe 0.71, Si 0.31, Zn 0.21, Mn 0.19, Cu 0.18, Cr 0.13, and Al balance. In the preparation stage, 27 experimental specimens were cut down by laser cutting. The dimensions of the specimens are shown in Figure 1. At the same time, argon and nitrogen were selected as processing gases to prevent edge oxidation. Compared with cutting methods such as traditional cutting and WEDM, high accuracy and fine cutting edges can be obtained by lasing cutting [21]. For the experiment, specimens were put into a resistance furnace heated to designated temperatures and kept for 10 minutes. Then, tensile tests were conducted at different strain rates until the specimens fractured; the strain was obtained with the help of a high-temperature extensometer for the whole process. The temperatures (633 K, 703 K, 773 K) in the tensile tests were above the recrystallization temperature because of the substantial improvement of plasticity [22]. Since the softening mechanisms (like dynamic recovery and dynamic recrystallization) that improve the processability are more fully carried out at low strain rates [23], low strain rates of 0.0003 s^−1^, 0.003 s^−1^, 0.03 s^−1^ in different orders of magnitude were adopted. Each test was repeated three times under a specified temperature and strain rate on the model MTS-810 tensile test machine. Then, the tests in nine different conditions were conducted. The standard deviation (*S*) was calculated in different experiment conditions:

(1) $$S=\frac{1}{m}\overset{m}{\underset{j}{\sum }}\sqrt{\frac{\sum_{i=1}^{n}{\left(\sigma_{i}-{\bar{\sigma }}_{i}\right)}^{2}}{n}},$$

where σ is the flow stress at the specified true strain in the specified experiment condition; $\bar{\sigma }$ is the average flow stress at the specified true strain in the specified experiment condition; *n* is the number of specified true strain points (603 points were selected with interval 0.0004); *m* is the number of repeat experiments (at 3).

**Figure 2:**
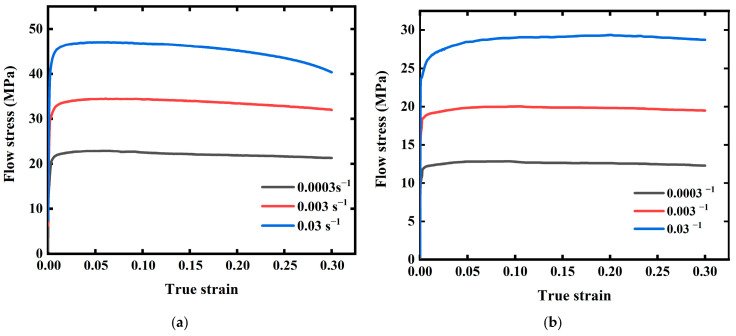

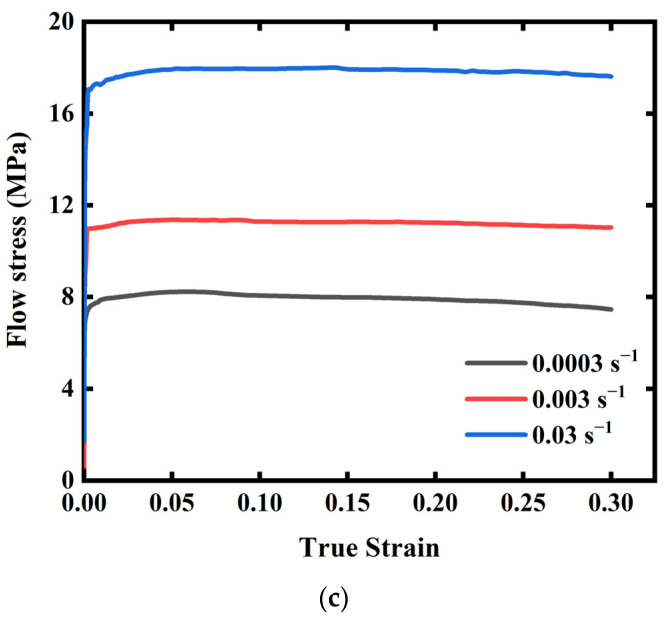
Flow stress-true strain at different strain rates in different temperatures. (**a**) 633 K. (**b**) 703 K. (**c**) 773 K.

Table 1 shows the experimental parameters of nine isothermal tensile tests and their *S* values with acceptable deviations. Hence, the average value of three repeat tests was adopted. Finally, flow stress-true strain curves in the strain range 0–0.3 were obtained and displayed in Figure 2.

### 2.2. Establishment of Arrhenius-Type Constitutive Equation

The concept that the variation of flow stress σ (MPa) depends on strain rate $\dot{\varepsilon }\left(s^{-1}\right)$ and temperature T (K) in the deformation process has been widely recognized. Research shows that relational expression equations among  σ, $\dot{\varepsilon }$, and *T* can be divided into the three conditions below [24,25]:

(1) At low stress (ασ<0.8):

(2) $$\dot{\varepsilon }=A_{1}\sigma^{n^{\prime }}\exp\left(-\frac{Q}{RT}\right),$$

(2) At high stress (ασ>1.2):

(3) $$\dot{\varepsilon }=A_{2}\exp\left(\beta \sigma \right)\exp\left(-\frac{Q}{RT}\right),$$

(3) For whole stress range:

(4) $$\dot{\varepsilon }=A{\left[\sinh\left(\alpha \sigma \right)\right]}^{n}\exp\left(-\frac{Q}{RT}\right),$$

where $Q\;\left(J\cdot {\mathrm{mol}}^{-1}\right)$ is the activation energy; $R\;\left(8.314\;J\cdot {\mathrm{mol}}^{-1}\cdot K^{-1}\right)$ is the gas constant; $A_{1}$, $A_{2}$, A, n, $n^{\prime }$, β, $\alpha \left(\approx \frac{\beta }{n^{\prime }}\right)$ are temperature independent material constants.

According to the work of Zener and Hollomon [26], the plastic deformation behavior under high temperatures was controlled by Q. Hence, the *Z* parameter considering the temperature compensated strain rate with the exponential function was invented to express the relation during plastic deformation. The relationship description between flow stress and strain rate under a wide temperature range was realized by the Zener-Hollomon model [11,12,13,14,15]:

(5) $$Z=\dot{\varepsilon }\exp\left(\frac{Q}{RT}\right),$$

where $Z\left(s^{-1}\right)$ is the Zener-Hollomon parameter, a strain rate factor for the temperature compensation.

The hyperbolic sine function is transformed by definition and combined Equation (4) with Equation (5), the equation defining the σ , $\dot{\varepsilon }$, and *T* relation can be written as follows:

(6) $$\sigma =\frac{1}{\alpha }\ln\left\{{\left(\frac{Z}{A}\right)}^{\frac{1}{n}}+{\left[{\left(\frac{Z}{A}\right)}^{\frac{2}{n}}+1\right]}^{\frac{1}{2}}\right\},$$

To achieve the purpose of parameter acquisition by expressing the relationship between σ and $\dot{\varepsilon }$ in a linear manner. The natural logarithms were taken from Equations (2)–(4), and after being converted, the new equations were obtained as follows:

(7) $$\ln\sigma =\frac{1}{n^{\prime }}\ln\dot{\varepsilon }-\frac{1}{n^{\prime }}\ln B,$$

(8) $$\sigma =\frac{1}{\beta }\ln\dot{\varepsilon }-\frac{1}{\beta }\ln C,$$

(9) $$\ln[\sinh\left(\alpha \sigma \right)]=\frac{\ln\dot{\varepsilon }}{n}+\frac{Q}{nRT}-\frac{\ln A}{n},$$

Based on Equations (7) and (8), and $\ln\sigma -\ln\dot{\varepsilon }$, $\sigma -\ln\dot{\varepsilon }$ curves, ten $n^{\prime }$ values and ten β values were calculated by taking average slopes of curves during strain range 0.03–0.3 with strain interval 0.03. Meanwhile, ten α values were also calculated by Equation $\alpha \approx \beta /n^{\prime }$.

The Equation (9) was partially differentiated to obtain the relation between σ and $\dot{\varepsilon }$, which leads to:

(10) $$Q=R{\left\{\frac{\partial \ln\dot{\varepsilon }}{\partial \ln\left[\sinh\left(\alpha \sigma \right)\right]}\right\}}_{T}{\left\{\frac{\partial \ln[\sinh\left(\alpha \sigma \right)]}{\partial \left(\frac{1}{T}\right)}\right\}}_{\dot{\varepsilon }},$$

Substituting different α values at ten strains to Equation (10). By combing with $\ln[\sinh\left(\alpha \sigma \right)]-\ln\dot{\varepsilon }$ curves and taking average slopes, ten *n* values at strain range 0.03–0.3 with 0.03 interval were obtained. Similarly, ten Q values were also calculated with the help of ln[sinh(ασ)]−ln(1/T) curves.

**Figure 3:**
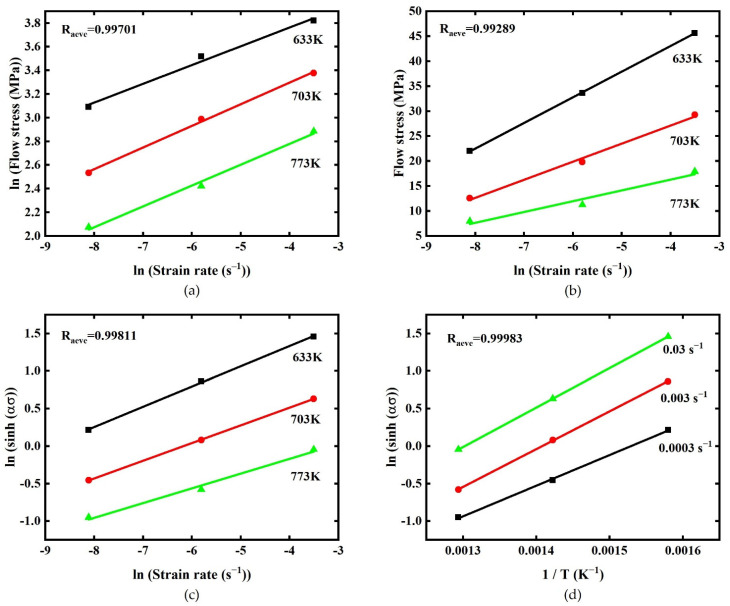
Relationships between (**a**) ln (Flow stress (MPa))-ln (Strain rate (s^−1^)), (**b**) Flow stress (MPa)-ln (Strain rate (s^−1^)), (**c**) ln (sinh (ασ))–ln (Strain rate (s^−1^)), ln (sinh (ασ))–1/*T* (K^−1^) (**d**) at the true strain 0.18.

The relationship between Z and σ can be directly established to obtain lnA values. Substituting Equation (4) to Equation (5) and taking both sides’ natural logarithm of the new Equation, Equation (11) was acquired after conversion:

(11) lnZ=nln[sinh(ασ)]+lnA,

Finally, by combining Equation (11) with the plot of lnZ−ln[sinh(ασ)], ten lnA values were obtained at ten strains during 0.03–0.3 with 0.03 strain interval. Take true strain 0.18 as example: Figure 3 shows the $\ln\sigma -\ln\dot{\varepsilon }$ (Equation (7)), $\sigma -\ln\dot{\varepsilon }$ (Equation (8)), $\ln[\sinh\left(\alpha \sigma \right)]-\ln\dot{\varepsilon }$ (Equation (10)), ln[sinh(ασ)]−ln (1/T) (Equation (10)) plots and the average correlation coefficients (R_aver_) are also showed in the figure. Thus far, ten α values, ten n values, ten Q values, and ten lnA values were obtained, which are shown in Table 2.

It is noteworthy that other than strain rate and deformation temperature, the true strain also plays a significant part during “flow stress change”. Nevertheless, the influence of true strain is not considered in the above equations. In former research, it has been widely recognized that α, n, Q, lnA parameters are also influenced by true strain and can be expressed by functions of true strain [27]. Hence, taking strain as an impact factor of α, n, Q, and lnA the universality of the equation under different strains will be improved. Polynomial function fitting is a practical method and has been acknowledged by researchers. At a specified strain, the average values of α, n, Q, and lnA can be calculated, then polynomial function curves can be adopted to describe the variations of α, n, Q, and lnA values at different true strains. Consequently, aided by the polynomial function, the goal of taking the strain into consideration is achieved. It is worth noticing that higher accuracy can be obtained by a greater polynomial order. This is because the higher the polynomial order, the higher the degree of freedom of fitting. However, it will also lead to the complexity of the solution process [28]. In this work, a six-order polynomial function (as presented by Equation (12)) was applied to fit α, n, Q, and lnA values, and the calculation results are shown in Table 2:

(12) $$\alpha =B_{0}+B_{1}\varepsilon +B_{2}\varepsilon^{2}+B_{3}\varepsilon^{3}+B_{4}\varepsilon^{4}+B_{5}\varepsilon^{5}+B_{6}\varepsilon^{6}\;n=C_{0}+C_{1}\varepsilon +C_{2}\varepsilon^{2}+C_{3}\varepsilon^{3}+C_{4}\varepsilon^{4}+C_{5}\varepsilon^{5}+C_{6}\varepsilon^{6}\;Q=D_{0}+D_{1}\varepsilon +D_{2}\varepsilon^{2}+D_{3}\varepsilon^{3}+D_{4}\varepsilon^{4}+D_{5}\varepsilon^{5}+D_{6}\varepsilon^{6}\;\ln A=E_{0}+E_{1}\varepsilon +E_{2}\varepsilon^{2}+E_{3}\varepsilon^{3}+E_{4}\varepsilon^{4}+E_{5}\varepsilon^{5}+E_{6}\varepsilon^{6}$$

where $B_{i}$, $C_{i}$, $D_{i}$*,* $E_{i}\;\left(i=0,\;1,\;2,\;\ldots ,\;6\right)$ denotes sixth-order polynomial coefficients.

**Figure 4:**
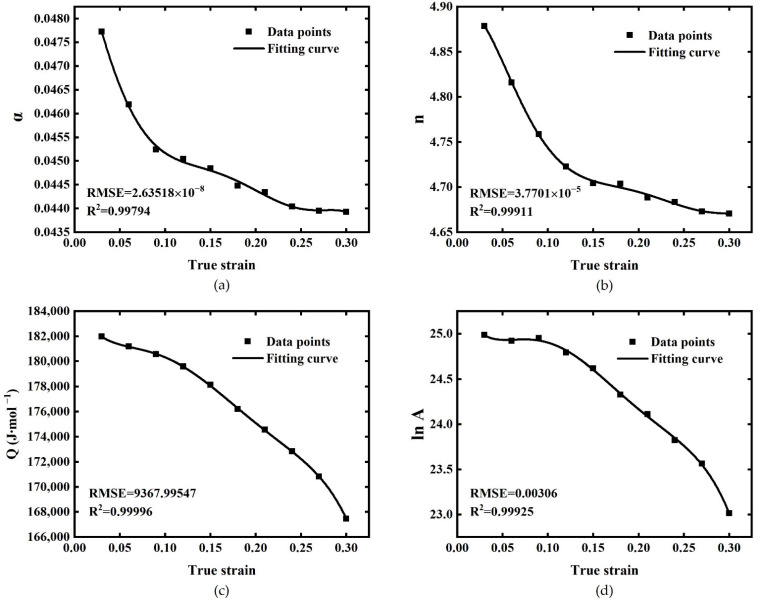
Fitting curves of (**a**) α, (**b**) n, (**c**) Q, and (**d**) lnA by the sixth-order polynomial.

The polynomial coefficients after fitting are shown in Table 3, and the results of fitting curves were shown in Figure 4. The RMSEs of α, n, Q, lnA are 2.63518 × 10^−8^, 3.7701 × 10^−5^, 9367.99547, 0.00306 and all R^2^ values of four parameters are above 0.99, which means that the sixth order polynomial function can fit these data with enough accuracy.

In summary, substituting Equation (12) to Equation (6), an Arrhenius-type equation integrated into the influence of strain compensation was obtained to describe AA5005 alloy flow behavior in the 0.03–0.3 strain range, 0.0003–0.03 s^−1^ strain rate range, and 633–773 K temperature range. Equation (13) expresses the final form of the Arrhenius-type Equation:

(13) $$\sigma =\frac{1}{\alpha \left(\varepsilon \right)}\ln\left\{{\left(\frac{Z^{\prime }}{A\left(\varepsilon \right)}\right)}^{\frac{1}{n\left(\varepsilon \right)}}+{\left[{\left(\frac{Z^{\prime }}{A\left(\varepsilon \right)}\right)}^{\frac{2}{n\left(\varepsilon \right)}}+1\right]}^{\frac{1}{2}}\right\}\;Z^{\prime }=\dot{\varepsilon }\exp\left[\frac{Q\left(\varepsilon \right)}{RT}\right]$$

where $Z^{\prime }$ is the Zener-Hollomon parameter considering the effect of the true strain.

### 2.3. Modeling by BP-ANN Model

From the theory Werbos [16] put forward that applies the BP algorithm to the ANN, Rumelhart et al. [29] explained the internal connection can be expressed by a hidden layer that applies to input data. Next, the new machine learning methods like SVM, LeNet, and AlexNet were proposed by Cortes C, LeCun, and Alex, respectively [30,31,32]. Essentially, the BP-ANN’s input–output relationship is a highly nonlinear continuous mapping relation. Additionally, the information processing capability of the BP-ANN origins from multiple compositions of simple nonlinear functions, and therefore it has a strong function reproduction ability.

The BP-ANN model is a feed-forward neural network adding to the error back propagation algorithm training. This model owns the classification ability of arbitrary complex patterns and excellent mapping ability of multi-dimensional functions [33]. Structurally, one input layer, one output layer, and the optional number of hidden layers are included in one ANN model. Each structure layer is composed of a certain number of neurons; in principle, under the premise that mean square error (MSE) was taken as the objective function, the BP algorithm calculates the minimum value of the objective function with the help of the gradient descend updating method.

**Figure 5:**
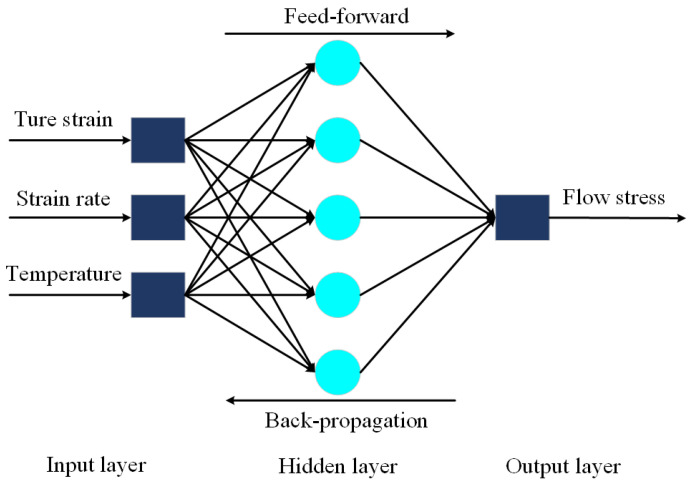
Structure schematic BP-ANN model of the present work.

In the input layer, true strain ε, strain rate $\dot{\varepsilon }$ (s^−1^), and temperature *T* (K) were set as three input neurons. One hidden layer with five built-in neurons was adopted in the present work. In the output layer, just the flow stress σ (MPa) as one output neuron was set, as can be seen in Figure 5. For data training, 603 sets of data were selected from experimental data. Among them, 70% of the whole sets (422) were chosen randomly to be trained, and the remaining 30% of the sets (181) were divided equally to be used to validate and test the BP-ANN model, respectively.

Before the training work, the normalization work needs to be carried out with input experimental values for machine-reading. A commonly used equation for normalizing the data into the 0–1 range is presented as [34]:

(14) $$e^{\prime }=\frac{e-0.95e_{min}}{1.05e_{max}-0.95e_{min}}\;,$$

where e is the experimental value; $e_{min}$ is the minimum value of experimental values; $e_{max}$ is the maximum value of experimental values; $e^{\prime }$ is the normalized value of e.

During the process of network operation, all data going through neurons should be assisted with activation functions. The activation functions are used to introduce nonlinear factors to solve the nonlinear problems [35]. Since the easily differential property of hyperbolic and linear functions, two corresponding activation functions (sigmoid function and purlin function) were adopted by hidden layer neurons and output layer neurons as activation functions, respectively:

(15) $$\mathrm{Sigmoid}:f\left(x\right)=\frac{1}{1+\exp\left(-x\right)}\;\;\mathrm{Purelin}:f\left(x\right)=x$$

In the process of feed-forward propagation. The propagation formulas of hidden layer neurons and output layer neurons are as follows:

(16) $$u_{j}=f\left(\overset{n}{\underset{i=1}{\sum }}v_{ij}x_{i}+\theta_{j}^{u}\right)\;p=f\left(\overset{m}{\underset{j=1}{\sum }}w_{j}u_{j}+\theta^{p}\right)$$

where $u_{j}$ is the output value of hidden layer neuron; *n* is the number of input layer neurons; $v_{ij}$ is the weight of input layer to hidden layer neuron; $x_{i}$ is the input value of input layer neuron; $\theta_{j}^{u}$ is the bias of hidden layer neuron; p is the output value of output layer neuron; *m* is the number of hidden layer neurons; $w_{j}$ is the weight of hidden layer neuron to output layer neuron; $\theta^{P}$ is the bias of output layer neuron.

To measure the deviation between the input experimental value and predicted value, the MSE was used at the end of each iteration during the back-propagation process:

(17) $$E_{MSE}=\sqrt{\sum_{i=1}^{N}\frac{{\left(e_{i}-p_{i}\right)}^{2}}{N}},$$

where *N* is the training times (20), *e* is the experimental value, and *p* is the actual neuron output of the output layer (predicted value) after feed-forward propagation.

Matching to the Widrow-Hoff learning rule based on the gradient descent, the updated algorithm was employed for initializing bias and weight [36]. To explain the gradient descent update algorithm, the training error must be introduced. Thus, the training error is the quadratic function of the input weight and bias. The gradient vector can be obtained by taking the partial derivative of the weight and bias of the training error, respectively. The opposite direction of the gradient vector is the direction of the gradient decreasing the fastest, and it is easier to find the minimum value of training error in this direction. However, the local optimum problem may happen by using the gradient descent update algorithm. To prevent this problem and improve the model generalization ability, the maximum iterative time was set as 40 and the number of generalization ability checking was set as 6 [37]. In the network, the parameters $v_{ij}$, $\theta_{j}^{u}$, $w_{j}$, and $\theta^{p}$ can be optimized by gradient descent update algorithm:

(18) $$\overset{ˇ}{w}=w-u\frac{dy}{dw}\;,$$

where $\overset{ˇ}{w}$ is the optimized parameter; w is the parameter to be optimized ($v_{ij}$, $\theta_{j}^{u}$, $w_{j}$, $\theta^{p}$); y is the training error; u is descend rate (learning rate).

Substituting $v_{ij}$, $\theta_{j}^{u}$, $w_{j}$, $\theta^{y}$ into Equation (18) and combing the obtained equation with Equation (15), the back-propagation gradient descent equation in the present work can be expressed as follows:

(19) $$\frac{\partial J^{\left(k\right)}}{\partial v_{ij}}=2\left(e_{k}-p_{k}\right)w_{j}u_{j}\left(1-u_{j}\right)x_{i}^{\left(k\right)}\;\frac{\partial J^{\left(k\right)}}{\partial \theta_{j}^{u}}=2\left(e_{k}-p_{k}\right)w_{j}u_{j}\left(1-u_{j}\right)\;\frac{\partial J^{\left(k\right)}}{\partial w_{j}}=2\left(e_{k}-p_{k}\right)u_{j}\;\frac{\partial J^{\left(k\right)}}{\partial \theta^{y}}=2\left(e_{k}-p_{k}\right)$$

where k is the number of experimental input–output data sets for training (k∈[1, 422]).

**Figure 6:**
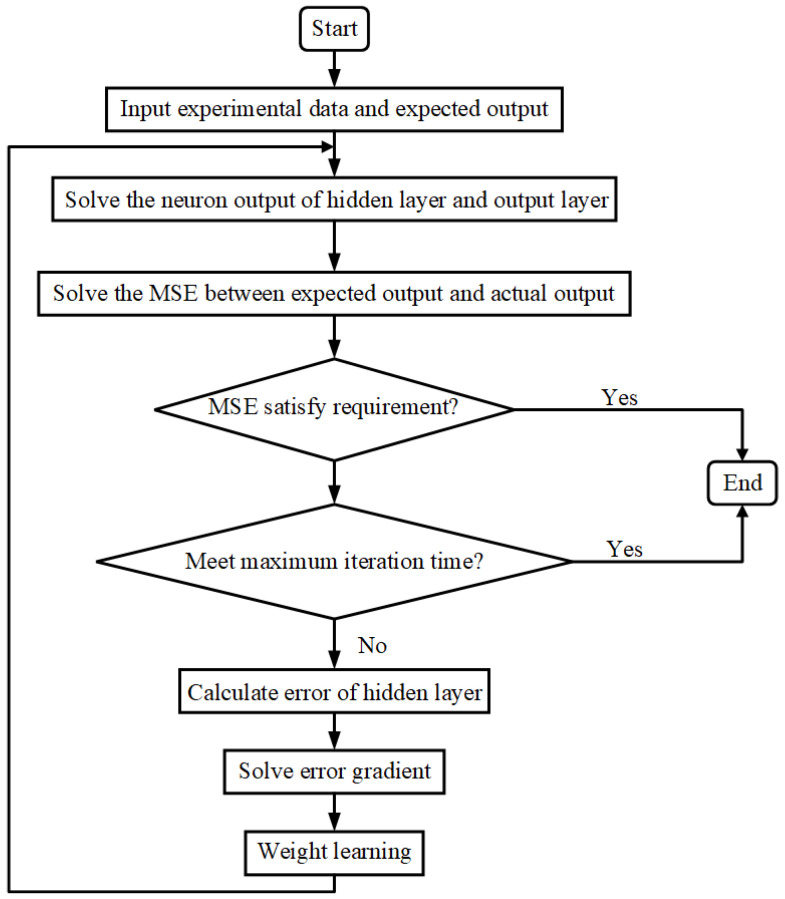
Flow chart of the BP-ANN model.

Based on Equations (13)–(19), the BP-ANN model program enters the iterative cycle until the MSE value satisfies the model requirement or the maximum training time reaches the maximum number. The flow chart of the BP-ANN model is given in Figure 6.

## 3. Results

**Figure 7:**
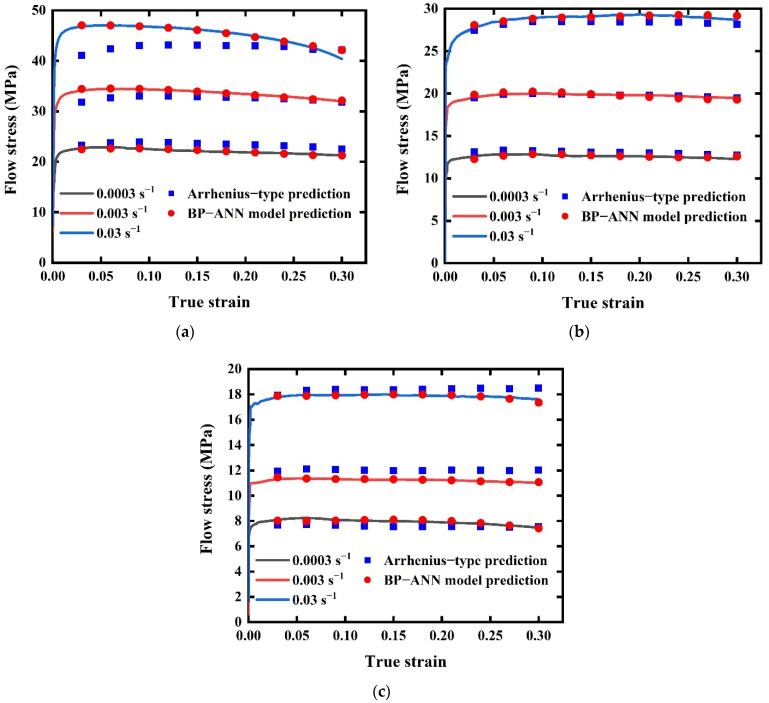
Comparison plots among experimental curves; values predicted by the Arrhenius-type equation and values predicted by the BP-ANN model. (**a**) 633 K. (**b**) 703 K. (**c**) 773 K.

After 30 iterations, BP-ANN model training stopped with MSE 0.0266 and gradient 0.742. Table 4 shows the R^2^ values of training data, validation data, test data, and all data of the BP-ANN model. All R^2^ values were very close to 1, which indicates the predicted values of the ANN-model had great correlation. Figure 7 shows the comparisons of the experimental curves and predicted values by the Arrhenius-type equation and BP-ANN model at each strain rate (0.0003 s^−1^, 0.003 s^−1^, 0.03 s^−1^) and temperature (633 K, 703 K, 773 K). It can be seen that the Arrhenius-type equation can predict flow stress with true strain change in most deforming conditions, except at stain rate 0.03 s^−1^ and temperature 633 K. However, almost all the points predicted by the BP-ANN model are located on the experimental curves, showing higher accuracy.

## 4. Discussion

From Figure 7, a large deviation occurs at 0.03 s^−1^ strain rate while flow stress is high. This indicates that the prediction capability of the Arrhenius-type regression equation will fail to meet the requirement when high accuracy is needed. To explain this phenomenon, we should combine Equations (2) and (3) with α values calculated before. It was found that all ασ values in this study were lower than 0.8, which means all tensile tests were carried out under low stress (ασ < 0.8). Therefore, the developed Arrhenius-type model may lose accuracy in high-stress ranges.

To comparatively investigate the predictability of both models, the statistical measurement analyses R^2^, AARE, and relative error (δ) were adopted during this study. The R^2^ is used to verify the correlation between experimental values and predicted values. The closer the R^2^ value is to the critical correlation coefficient (present work: 1), the higher the correlation. However, because the bias is not considered, the R^2^ value is insufficient to verify the accuracy. Nevertheless, the AARE makes up for the above shortcoming by calculating the relative error between the predicted value and experimental value, term by term. The smaller the AARE value, the more accurate the predicted value is. Consistently, δ is employed for observing error distribution on most occasions. With more data concentrated in the area with small δ values, the model with better predicting performance can be obtained. The R*^2^*, the AARE, and the δ can be calculated by the following three equations [38,39,40]:

(20) $$R^{2}=\frac{\sum_{i=1}^{N}\left(E_{i}-\bar{E}\right)\left(P_{i}-\bar{P}\right)}{\sqrt{\sum_{i=1}^{N}{\left(E_{i}-\bar{E}\right)}^{2}\sum_{i=1}^{N}{\left(P_{i}-\bar{P}\right)}^{2}}}\;,$$

(21) $$AARE=\frac{1}{N}\overset{N}{\underset{i=1}{\sum }}\left|\frac{E_{i}-P_{i}}{E_{i}}\right|\times 100\%\;,$$

(22) $$\delta =\frac{E_{i}-P_{i}}{E_{i}}\times 100\%\;,$$

where *E* is the experimental flow stress value; $\bar{E}$ is the average value of experimental flow stress values; *P* is the predicted flow stress value; $\bar{P}$ is the average value of predicted values.

**Figure 8:**
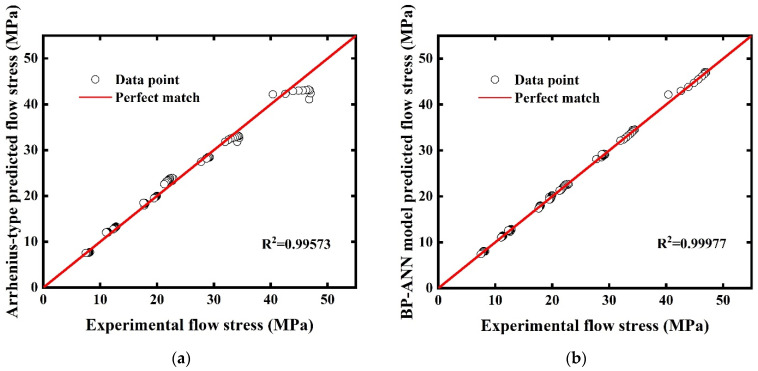
Correlation plots of experimental flow stress values and predicted values. (**a**) Arrhenius-type equation. (**b**) BP-ANN model.

The correlation strength can be expressed by correlation plots. If a predicted value is closer to the experimental value, the corresponding point will be closer to the perfect match line. Figure 8a shows flow stress values predicted by the Arrhenius-type equation are well satisfied with a linear relationship with R^2^ 0.99573. Large deviations occur at the high-stress stage, which is in accordance with the phenomenon we discussed before. However, in Figure 8b, almost all data points are distributed on the perfect match line and with R^2^ 0.99977, which demonstrates an excellent linear relationship was achieved by the BP-ANN model. Instead of other models having specific formula expressions, the working mechanism of the BP-ANN model is more like a black box that adjusts the weights and biases automatically, like neurons in the human body. The BP-ANN model, which achieves the target accuracy through multiple iterations, fits the experimental data better than the Arrhenius model, only through finite linear regressions.

**Figure 9:**
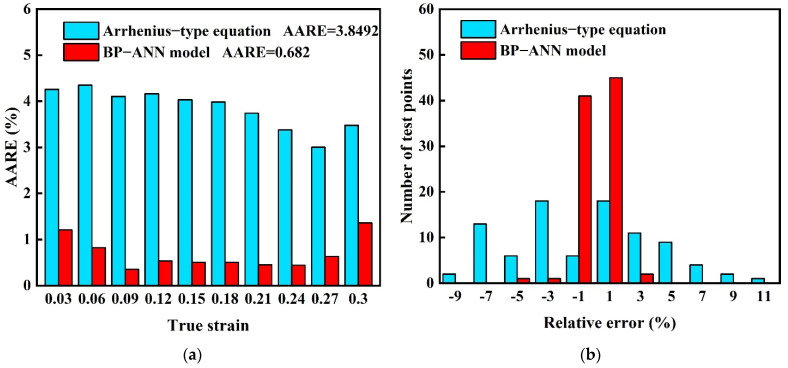
Statistical analyses: (**a**) AAREs for two models at different true strains and (**b**) relative error distribution of two models.

In order to make a comparative study of AAREs at different strains within the experimental strain range, histograms were created for a visual representation, see Figure 9a. The AAREs between the Arrhenius-type predicted values and experimental data at all kinds of strains are above 3%. However, the AAREs of the BP-ANN model at all strains are all under 1.5%, which is much lower than that of the Arrhenius-type equation. By combining the comparison of the whole strain range AAREs (Arrhenius-type equation 3.8492 and BP-ANN model 0.682), the conclusion could be reached that the BP-ANN model always keeps lower deviations than the Arrhenius-type equation in the whole strain range. Furthermore, another obvious comparison can be seen from the relative error distribution histogram of the two models (Figure 9b). A large range of relative error (−9–11%) is occupied by the Arrhenius-type equation, and distribution is relatively scattered, which indicates the Arrhenius-type equation’s low reliability. On the contrary, embracing a narrow error range (−5–3%) and that 95% of errors are located in the range −1–1%, the BP-ANN model is reliable to maintain a low accuracy.

**Figure 10:**
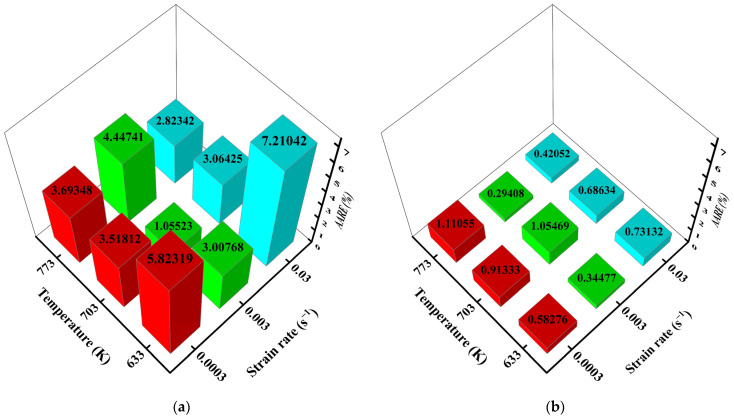
3D histograms of AAREs at different conditions of (**a**) Arrhenius-type equation and (**b**) BP-ANN model.

By calculating the AAREs of ten scatter points (see Figure 7) at a specified true strain but in different deformation conditions (different temperatures and strain rates), the AARE distributions were analyzed with the help of 3D histogram plots, see Figure 10. As in Figure 10a, large errors emerge in the working conditions that cause high flow stress values. For example, the 7.21042% AARE value can be discovered at strain rate 0.03 s^−1^, temperature 633 K. This phenomenon also shows consistency with Figure 7a and Figure 8a. In general, AAREs of the Arrhenius-type equation keep high values, except at strain rate 0.003 s^−1^ and temperature 703 K. At the same time, the BP-ANN model’s AAREs are much smaller than that of the Arrhenius-type equation, and no big AARE occurs under all conditions, see Figure 10b. Especially in the case of high stress, the accuracy of the BP-ANN model is not affected. Hence, the prediction ability of the BP-ANN model is better than the Arrhenius-type regression equation under most of the working conditions proposed in this paper.

## 5. Conclusions

High temperature and low strain rate tensile tests were conducted to obtain the flow stress curves of aluminum AA5005 alloy in the 0–0.3 strain range, 0.0003–0.03 s^−1^ strain rate range, and 603–773 K deformation temperature range. In this study, a strain-compensated Arrhenius-type constitutive equation and BP-ANN model were adopted to establish constitutive relation. Additionally, the calculating processes of the two models were expounded exhaustively in this work. Following are the conclusions:

(1) By scattering predicted points of two models to compare with the experimental flow stress-true strain curves, both models can describe the flow behavior of AA5005 alloy. However, drawbacks were discovered from the comparison plots and statistical analyses. The results show the Arrhenius-type equation is disabled to predict the flow behavior at high flow stress conditions. The reason for this phenomenon is that all tests were processed in low flow stress, which is a little incompatible with the Arrhenius-type equation considering overall flow stress.

(2) Furthermore, the correlations and accuracy of two models in different strains were compared. Both models keep excellent correlations with the R^2^ values 0.99573 and 0.99977, respectively. In terms of AARE analysis, in different strains, the performance of the BP-ANN model is far better than the Arrhenius-type model. In different deform conditions, the BP-ANN model also has better accuracy (except at strain rate 0.003 s^−1^ and temperature 703 K). Comparing the overall AAREs of the two models, the BP-ANN model (0.682%) behaves much better than the Arrhenius-type equation (3.8492%).

(3) To make a comprehensive comparison, the relative error was also used to evaluate the models’ accuracy. The relative errors of the BP-ANN model are distributed in a more narrow and higher accuracy range than the Arrhenius-type equation. This means the BP-ANN model is more accurate and reliable than the Arrhenius-type equation in describing the flow behavior of AA5005 alloy.

The models developed in the present experiment lay a foundation for industrial weight-losing application and numerical simulation of the alloy under high-temperature and low strain rate tensile conditions. High accuracy can especially be achieved by the BP-ANN model.

## Figures and Tables

**Table 1:** Experimental parameters of tensile tests.

Test No.	Strain Rates (s^−1^)	Temperature (K)	Standard Deviation
1	0.0003	633	0.0146
2	0.003	633	0.0159
3	0.03	633	0.0173
4	0.0003	703	0.0091
5	0.003	703	0.0121
6	0.03	703	0.0148
7	0.0003	773	0.0073
8	0.003	773	0.0117
9	0.03	773	0.0153

**Table 2:** Values of *α*, *n*, *Q*, ln*A* at strains range 0.03–0.3 with interval 0.03.

Ture Strain	α	*n*	*Q* (J·mol^−1^)	ln*A*
0.03	0.04772	4.87868	181,964.43806	24.9859
0.06	0.04619	4.81612	181,175.14546	24.92136
0.09	0.04524	4.75863	180,574.88746	24.94958
0.12	0.04504	4.72281	179,593.61103	24.79107
0.15	0.04484	4.70455	178,120.69435	24.61697
0.18	0.04448	4.70354	176,220.69435	24.32659
0.21	0.04434	4.68823	174,562.29940	24.11042
0.24	0.04404	4.68339	172,830.64202	23.82443
0.27	0.04395	4.67296	170,823.64202	23.56193
0.30	0.04393	4.67052	167,461.87983	23.01320

**Table 3:** Polynomial coefficients of sixth-order polynomial function.

Polynomial Order	α	*n*	*Q* (J·mol^−1^)	ln *A*
0	0.04999	4.88046	184,672.26693	25.45296
1	−0.08123	2.193	−150,623.62814	−29.35556
2	0.01167	−103.62894	2.63224 × 10^6^	605.05764
3	7.56052	1096.26352	−2.30957 × 10^7^	−5582.67267
4	−60.0279	−5254.61372	8.83231 × 10^7^	23,224.34498
5	182.34278	11,975.12441	−1.39543 × 10^8^	−43,220.22732
6	−197.18793	−10,531.66483	5.25777 × 10^7^	26,822.76943

**Table 4:** R^2^ values of training data, validation data and test data of BP-ANN model.

	Training	Validation	Test	All
R^2^	0.99967	0.99982	0.99979	0.9997

## Data Availability

The data presented in this study are available on request from the corresponding author. The data are not publicly available due to these data are part of ongoing research.
